# Supplementary material for: Cerebellar nuclei cells produce distinct pathogenic spike signatures in mouse models of ataxia, dystonia, and tremor
Source: eLife. 2024 Jul 29;12:RP91483. doi: 10.7554/eLife.91483 (PMC11286262; doi:10.7554/eLife.91483)
Supplement: Supplementary file 1. — Each model used in this article is listed in the table with the type of model, that is, the predominant phenotype reported for the model, motor behaviors that have been quantified relative to control animals (effect direction is noted when difference is statistically significant), and anatomical changes associated with the model. Only congruent and/or undisputed findings in mice are included. Abbreviations used: Purkinje cell (PC), vesicular GABA transporter (Slc32a1), immunohistochemistry (IHC), wheat germ agglutinin (WGA), tyrosine hydroxylase (TH), vesicular glutamate transporter 2 (Slc17a6), inferior olive (IO), electromyography (EMG), cerebellar nuclei (CN), spinocerebellar ataxia type 1 (SCA1), carbonic anhydrase-related protein 8 (Car8), granule cell (GC), climbing fiber (CF), mossy fiber (MF), lateral hypothalamus (LH), and not available (NA). [file elife-91483-supp1.docx]

| Mouse Model | Model Type | Predominant Phenotype | Quantified Motor Behavior | Anatomy |
| --- | --- | --- | --- | --- |
| *Pcp2^Cre^;Slc32a1^fl/fl^* | Cre-loxP constitutive genetic knockout | Ataxia | **Impaired**  **↓ Hind stride:**  Footprint analysis  **↓ Latency to fall:**  Rotarod  **↓ Tremor amplitude:**  Tremor monitor  (Brown et al., 2020; White et al., 2014) | **↓ PC SLC32A1:** IHC and in situ  **Abnormal cerebellar zones:** IHC and WGA tracing  **↑ Ectopic TH expression:** IHC  (White et al., 2014) |
| *Ptf1a^Cre^;Slc17a6^fl/fl^* | Cre-loxP constitutive genetic knockout | Dystonia | **Impaired**  **↓ Distance traveled:**  Open field  **↓ Latency to fall:**  Rotarod  **↑ Tremor amplitude:**  Tremor monitor  **↑ Dystonia rating:**  Dystonia rating scale  **↑ EMG power:**  EMG  **↑ Antagonist muscle co-contraction and over-contraction:** EMG  (Brown et al., 2022, 2023; Salazar Leon & Sillitoe, 2023; White & Sillitoe, 2017) | **↓ IO SLC17A6:** IHC and in situ  **Transiently thinner molecular layer:** IHC  (Salazar Leon & Sillitoe, 2023; White & Sillitoe, 2017) |
| Harmaline injection (control genotype) | Intraperitoneal injection  (pharmacological) | Tremor | **Impaired**  **↑ Tremor amplitude:**  Tremor monitor  **↑ EMG power:**  EMG  (Brown et al., 2020, 2023) | **↑ CN and IO cFos expression:** IHC  (Brown et al., 2020) |
| *Atxn1^154Q/+^* | SCA1 genetic knockin | Ataxia | **Impaired**  **↓ Latency to fall:**  Rotarod  **↓ Distance traveled:**  Open field  **↑ Slips**  Dowel Assay  (Coffin et al., 2023; Fryer et al., 2011; Watase et al., 2002) | **↓ Brain weight**  **↑ Nuclear inclusions:** IHC  **↓ Calbindin immunofluorescence:** IHC  **Thinner molecular layer:** IHC  **↓ PC count:** IHC  **↑ Abnormal morphology and degeneration of motor neurons:** H&E  **↑ Reactive astrocytosis:** IHC  (Coffin et al., 2023; Fryer et al., 2011; Orengo et al., 2018; Watase et al., 2002) |
| *Pdx1^Cre^;Slc17a6^fl/fl^* | Cre-loxP constitutive genetic knockout | Dystonia | **Impaired**  **↑ Hind sway:**  Footprint analysis  **↓ Latency to fall:**  Rotarod  **↑ Dystonia rating:**  Dystonia rating scale  **↑ High stepping gait:**  Horizontal ladder  (Lackey, 2021) | **↓ CF and MF SLC17A6:** IHC  **↓ LH *Slc17a6*:** in situ  (Lackey, 2021)  (Mangieri et al., 2018) |
| *Pcp2^Cre^;Ank1^fl/fl^* | Cre-loxP constitutive genetic knockout | Ataxia | **Impaired**  **↓ Hind stride,**  **↓ Hind stance,**  **↑ Hind sway:**  Footprint analysis  (Stevens et al., 2021) | **↓ PC Ank1:** IHC  **PC progressive abnormal morphology and degeneration in anterior and central zones:** IHC  **Thinner molecular layer in anterior and central zones:** IHC  (Stevens et al., 2021) |
| Ouabain infusion (control genotype) | Application to the surface of the cerebellum (pharmacological) | Dystonia | **Impaired**  **↑ Dystonia rating:**  Dystonia rating scale  (Fremont et al., 2014) | NA |
| *Car8^wdl/wdl^* | Spontaneous deletion mutation | Ataxia + Dystonia + Tremor | **Impaired**  **↓ Latency to fall:**  Rotarod    **↓ Print width,**  **↓ Print area:**  CatWalk  **↑ Hind and fore base-width,**  **↑ Hind sway:**  Footprint analysis  **↑ Slips**  Raised beam  **↓ Ambulatory activity:**  Open field  **↑ Tremor amplitude:**  Tremor monitor  **↑ Antagonist muscle co-contraction and over-contraction:** EMG  **Unimpaired**  Righting time, vertical rope climbing, limb clasping  (Jiao et al., 2005; Miterko et al., 2019, 2021; White et al., 2016; Zhou et al., 2022) | **↓ PC Car8:** IHC  **Delayed and abnormal cerebellar zones:** IHC and WGA tracing  **Thinner molecular layer:** IHC  **Transiently smaller cerebellum:** H&E  **Delayed GC proliferation:** IHC  **Transiently abnormal PC morphology:** IHC  **↑ Ectopic TH expression:** IHC  (Miterko et al., 2019; White et al., 2016) |
| *Car8^wdl/wdl^ +* propranolol injection | Spontaneous deletion mutation + intraperitoneal injection  (genetic + pharmacological) | Ataxia + Dystonia | **Impaired**  **↑ Hind sway:**  Footprint analysis  **Unimpaired**  Tremor, open field (movement time, movement episodes, ambulatory activity)  (Zhou et al., 2022) | NA |
